# Methionine cycle inhibition disrupts antioxidant metabolism and reduces glioblastoma cell survival

**DOI:** 10.1016/j.jbc.2025.108349

**Published:** 2025-02-25

**Authors:** Emma C. Rowland, Matthew D'Antuono, Anna M. Jermakowicz, Nagi G. Ayad

**Affiliations:** Georgetown University, Lombardi Comprehensive Cancer Center, Washington, District of Columbia, USA

**Keywords:** glioblastoma, lipid peroxidation, metabolism, metabolomics, methionine, mitochondria, oxidative stress

## Abstract

Glioblastoma (GBM) is a highly aggressive primary malignant adult brain tumor that inevitably recurs with a fatal prognosis. This is due in part to metabolic reprogramming that allows tumors to evade treatment. Therefore, we must uncover the pathways mediating these adaptations to develop novel and effective treatments. We searched for genes that are essential in GBM cells as measured by a whole-genome pan-cancer CRISPR screen available from DepMap and identified the methionine metabolism genes *MAT2A* and *AHCY*. We conducted genetic knockdown, evaluated mitochondrial respiration, and performed targeted metabolomics to study the function of these genes in GBM. We demonstrate that *MAT2A* or *AHCY* knockdown induces oxidative stress, hinders cellular respiration, and reduces the survival of GBM cells. Furthermore, selective MAT2a or AHCY inhibition reduces GBM cell viability, impairs oxidative metabolism, and shifts the cellular metabolic profile towards oxidative stress and cell death. Mechanistically, MAT2a and AHCY regulate spare respiratory capacity, the redox buffer cystathionine, lipid and amino acid metabolism, and prevent oxidative damage in GBM cells. Our results point to the methionine metabolic pathway as a novel vulnerability point in GBM.

Glioblastoma (GBM) is the most common malignant primary brain tumor in adults (1). Despite efforts to identify new treatments for GBM, prognosis is dismal with a survival rate of approximately 15 months following diagnosis (2). The standard of care involves tumor resection along with a combination of temozolomide (TMZ), radiation treatment, and tumor-treating fields (3). Unfortunately, recurrence is nearly inevitable even following maximum bulk tumor resection. Therefore, there is a need to identify novel mechanisms or actionable targets in GBM to circumvent or mitigate disease progression.

Treatments are ineffective in part due to the metabolic reprogramming that occurs in GBM cells (4). The Warburg effect is thought to be essential for GBM-directed energy production (5, 6), but evidence of primary GBM cells demonstrating a highly oxidative phenotype with negligible response to glycolysis inhibitors suggests that GBM ultimately relies on oxidative metabolism for maximal ATP production (7). To support oxidative phosphorylation (OXPHOS), GBM cells depend on amino acid metabolism to supply reducing equivalents to the electron transport chain (8, 9). ATP production inevitably generates reactive oxygen species (ROS); thus, antioxidant production is required to scavenge ROS and prevent oxidative damage. Only certain amino acids have demonstrated strong antioxidative capacity (10).

Several studies have indicated that amino acid metabolism is significantly altered in GBM to promote tumorigenesis and progression (11, 12, 13, 14). Of 32 human cancers in The Cancer Genome Atlas database, GBM exhibits the highest expression of cysteine and methionine-related genes (15). In particular, methionine is an essential amino acid and directly supports several key processes, including methylation, nucleotide biosynthesis, immune activation, lipid metabolism, and antioxidant production (16). GBM tumors are uniquely dependent on methionine metabolism as evidenced through tumor imaging using positron emission tomography, revealing considerable ^11^C-methionine uptake compared to normal brain tissue (17). A recent study identified that methionine metabolism is integral to treatment resistance in GBM, with an upregulation of methionine-related metabolite production in tumor tissue following radiation (18). Methionine dependency has been further supported by reduced GBM growth through methionine media depletion *in vitro* and dietary methionine restriction *in vivo* (19, 20, 21). Additionally, methionine restriction has been shown to improve the efficacy of TMZ in an orthotopic mouse model (22). However, the mechanism by which this occurs and the process through which methionine promotes GBM tumor progression is not well understood.

Here, we identify two enzymes involved in methionine metabolism, MAT2a and AHCY, which are essential for GBM growth and redox balance. No known studies to date have interrogated these two enzymes and their involvement in GBM antioxidant metabolism. We demonstrate that both MAT2a and AHCY are required for proper mitochondrial function in GBM and are necessary to protect cancer cells against oxidative damage. Thus, our study designates these two metabolic enzymes as important candidates for targeted therapy in GBM.

## Results

### Genes encoding MAT2a and AHCY are essential in GBM and other CNS tumors

**Figure 1:**
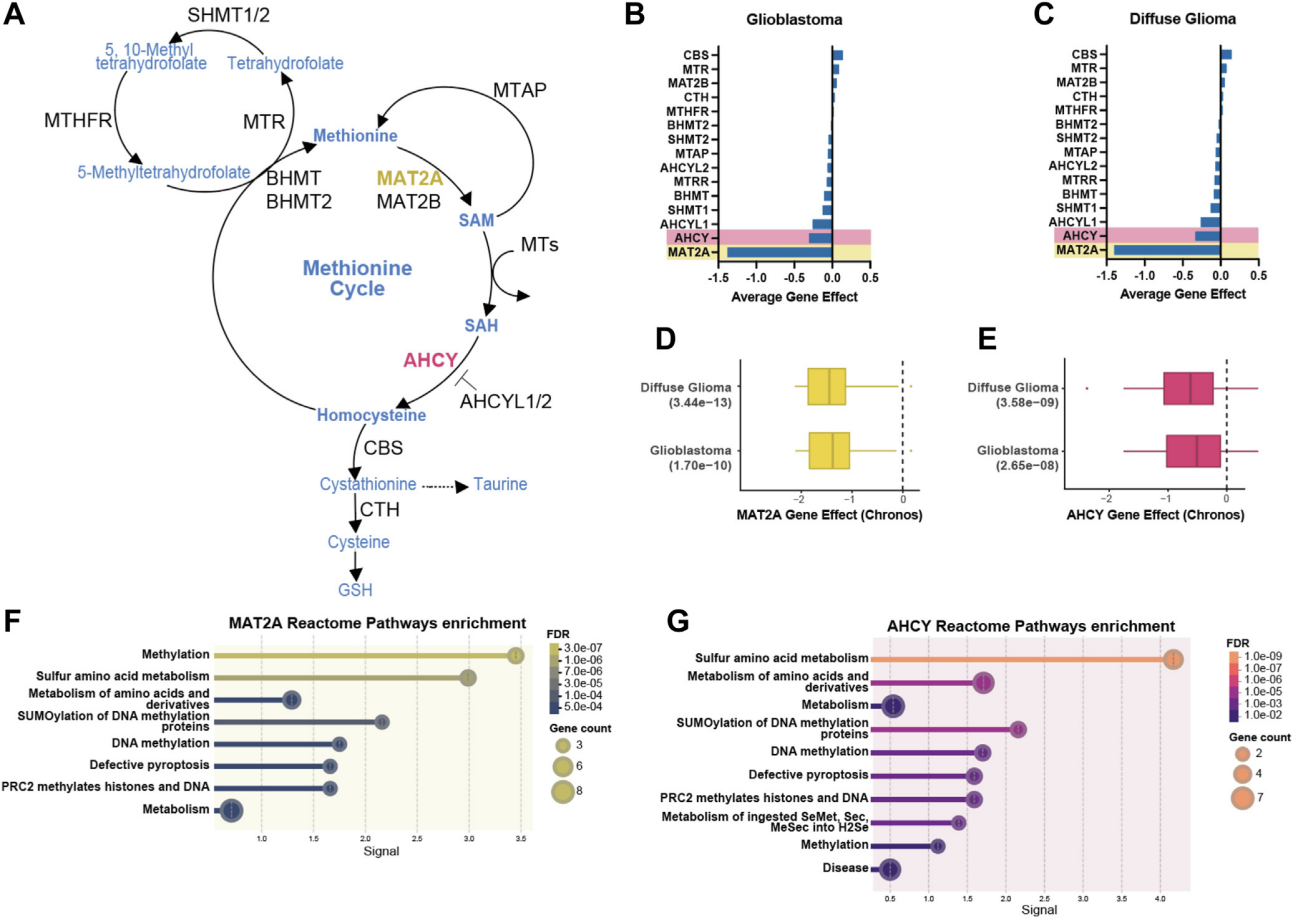
**Pooled CRISPR screen data reveals methionine metabolism gene essentiality in GBM.***A*, the methionine cycle contains multiple enzymes to generate methyl donor SAM and downstream antioxidant substrates. *B* and *C*, average gene effect scores representative of DepMap pooled CRISPR screens for indicated genes involved in the methionine cycle in glioblastoma and diffuse glioma lineages. Less than 0 indicates impaired cell growth and greater than 0 indicates enhanced cell growth. *D* and *E*, gene effect scores for both MAT2A and AHCY in diffuse glioma and glioblastoma lineages represented as boxplots scores filtered using *p*-value <0.00005 according to DepMap significance. *F* and *G*, significantly enriched pathways from the Reactome database for both MAT2A and AHCY, reflecting high signal for sulfur amino acid metabolism, generated using the STRING database.

We sought to identify whether specific methionine cycle–related metabolic genes are essential in GBM cell growth and survival. To explore this, we probed the Cancer Dependency Map or DepMap (https://depmap.org/portal) (23). DepMap is an online public database provided by the Broad Institute that utilizes large-scale functional genomics profiling in thousands of cancer cell lineages to elucidate gene essentiality. Their findings are based on pooled whole genome CRISPR-Cas9 screens across multiple cell lineages, primary diseases, and disease subtypes. A gene is deemed essential based on a calculated dependency score: an effect score of less than zero indicates reduced cell growth while an effect score of −0.5 or lower indicates induced cell death upon gene knockout. We investigated 15 primary metabolic genes involved in the methionine pathway in the DepMap database (Fig. 1, *A*–*C*). From this analysis, we identified two genes, *MAT2A* and *AHCY*, that upon genetic knockout were associated with reduced cell growth for diffuse glioma (*MAT2A* gene effect −1.4, *p* = 3.4 × 10^−13^; *AHCY* gene effect −0.335, *p* = 3.6 × 10^−9^) and GBM (*MAT2A* gene effect −1.38, *p* = 1.7 × 10^−10^; *AHCY* gene effect −0.305, *p* = 2.7 × 10^−8^) cell lineages (Fig. 1, *D* and *E*). *MAT2A* encodes the methionine adenosyltransferase (MAT2a) enzyme that directly utilizes methionine to generate the universal methyl donor S-adenosylmethionine (SAM), which is used by methyltransferases for histone and DNA modification (24). *AHCY* encodes the adenosylhomocysteinase (AHCY) enzyme that reversibly converts the unmethylated byproduct S-adenosylhomocysteine (SAH) to generate homocysteine and adenosine (25). *MAT2A* dependency was also found for other lineages including breast cancer, pancreatic cancer, and Ewing sarcoma (Fig. S1*A*). Other cell lines demonstrating significant *AHCY* dependency were associated with immune system cancers (Fig. S1*B*). In total, 71 central nervous system (CNS) cell lines showed significant dependency for *MAT2A* (mean gene effect −1.273 ± 0.407) and 36 showed significant dependency for *AHCY* (mean gene effect −0.680 ± 0.143), with 35 CNS cell lines exhibiting significant dependency for both genes (Fig. S2, *A* and *B*). Reactome pathway enrichment analyses were conducted using the STRING database (https://string-db.org) (26, 27) and indicated that sulfur amino acid metabolism was significantly enriched for both *MAT2A* and *AHCY* (Fig. 1, *F* and *G*). Sulfur is derived from methionine, and its metabolism constitutes the cellular antioxidant system responsible for maintaining redox homeostasis (28). Pathway enrichment and *MAT2A* and *AHCY* genetic dependency collectively suggest that these two genes and their encoded enzymes are candidates for targeting redox balance and cell growth in GBM.

### siRNA-induced knockdown of *MAT2A* and *AHCY* promotes cell death and lipid peroxidation in GBM

**Figure 2:**
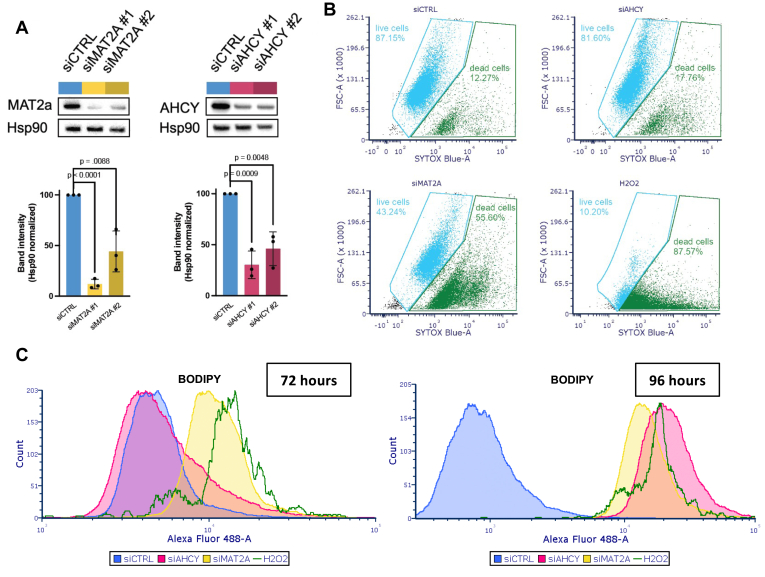
**Genetic knockdown of *MAT2A* and *AHCY* induces cell death and lipid peroxidation in GBM.***A*, gene knockdown in LN229 cells 48 h post-transfection, with heat shock protein 90 (Hsp90) as the loading control. Blots were quantified using ImageJ and normalized to siCTRL band intensity. Individual band intensity values represent biological replicates. Unpaired *t* test with Holm-Šídák correction was performed. *B*, cells stained with SYTOX Blue for flow cytometry analysis of cell death 96 h post-transfection, with hydrogen peroxide as positive control. *C*, BODIPY staining for flow cytometry analysis of lipid peroxidation 72 h and 96 h post-transfection as indicated on the graph. Cell counts were normalized to peak intensity, with hydrogen peroxide as positive control.

Based on these significant genetic dependency scores and pathway enrichments, we wanted to confirm the CRISPR screen results and further evaluate the role of *MAT2A* and *AHCY* in GBM cell survival and redox balance. We used selective siRNAs targeting either *MAT2A* or *AHCY* to evaluate the effect of gene silencing on LN229 cell survival. MAT2a and AHCY protein expression was significantly reduced (siMAT2A #1 11.98 ± 4.72%, p < 1 × 10^−4^; siMAT2A #2 44.18 ± 20.25%, *p* = 8.8 × 10^−3^; siAHCY #1 30.31 ± 13.59%, p = 9 × 10^−4^; 46.04 ± 16.53%, *p* = 4.8 × 10^−3^ compared to control % band intensity) at 48 h post-transfection (Figs. 2*A*; S3, *A*–*F*). Flow cytometry analysis revealed that there was a significant reduction in percent survival at 96 h after *MAT2A* knockdown (mean 53.733 ± 19.409% dead cells) compared to control (*p* = 0.0199) (Figs. 2*B*; S4*C*).

Lipid peroxidation is a process that occurs at the lipid membrane due to ROS disrupting lipid integrity (29). Cells rely on antioxidant protection to attenuate lipid peroxidation (30). Compared to nonmalignant cells, cancer cells have higher levels of lipid peroxidation that support their growth and survival (31). In GBM, lipid peroxidation has been observed at the invasive front of the tumor and is found to facilitate immune and therapeutic resistance (32). However, excess lipid peroxidation leads to compromised function and ferroptotic cell death (33), and this has been previously linked to methionine restriction in GBM (21). Upon *MAT2A* knockdown, we observed an increase in lipid peroxidation (BODIPY) staining at 72 h (Figs. 2*C*; S4*B*). After 96 h, we observed a further increase in lipid peroxidation after *MAT2A* knockdown as well as increased lipid peroxidation staining after *AHCY* knockdown cells compared to controls (Figs. 2*C*; S4*C*). These results confirm that the GBM cell line LN229 is dependent on *MAT2A* for survival and suggest that *MAT2A* or *AHCY* are required to evade oxidative damage in GBM.

### *MAT2A* and *AHCY* knockdown compromises mitochondrial function in GBM

We next sought to investigate the oxidative metabolism changes associated with *MAT2A* and *AHCY*. The primary function of mitochondria is to supply energy to cells through OXPHOS (34). They contribute two primary sources of ROS generation during OXPHOS *via* the electron transport chain complexes I and III (35). It is well appreciated that maintaining ROS at appropriate levels is necessary for normal cell function (36), but elevated levels of ROS can be activating for cancer proliferation, invasion, and metastasis in GBM (37, 38, 39). Therefore, we identified key parameters of mitochondrial bioenergetics following genetic knockdown.

**Figure 3:**
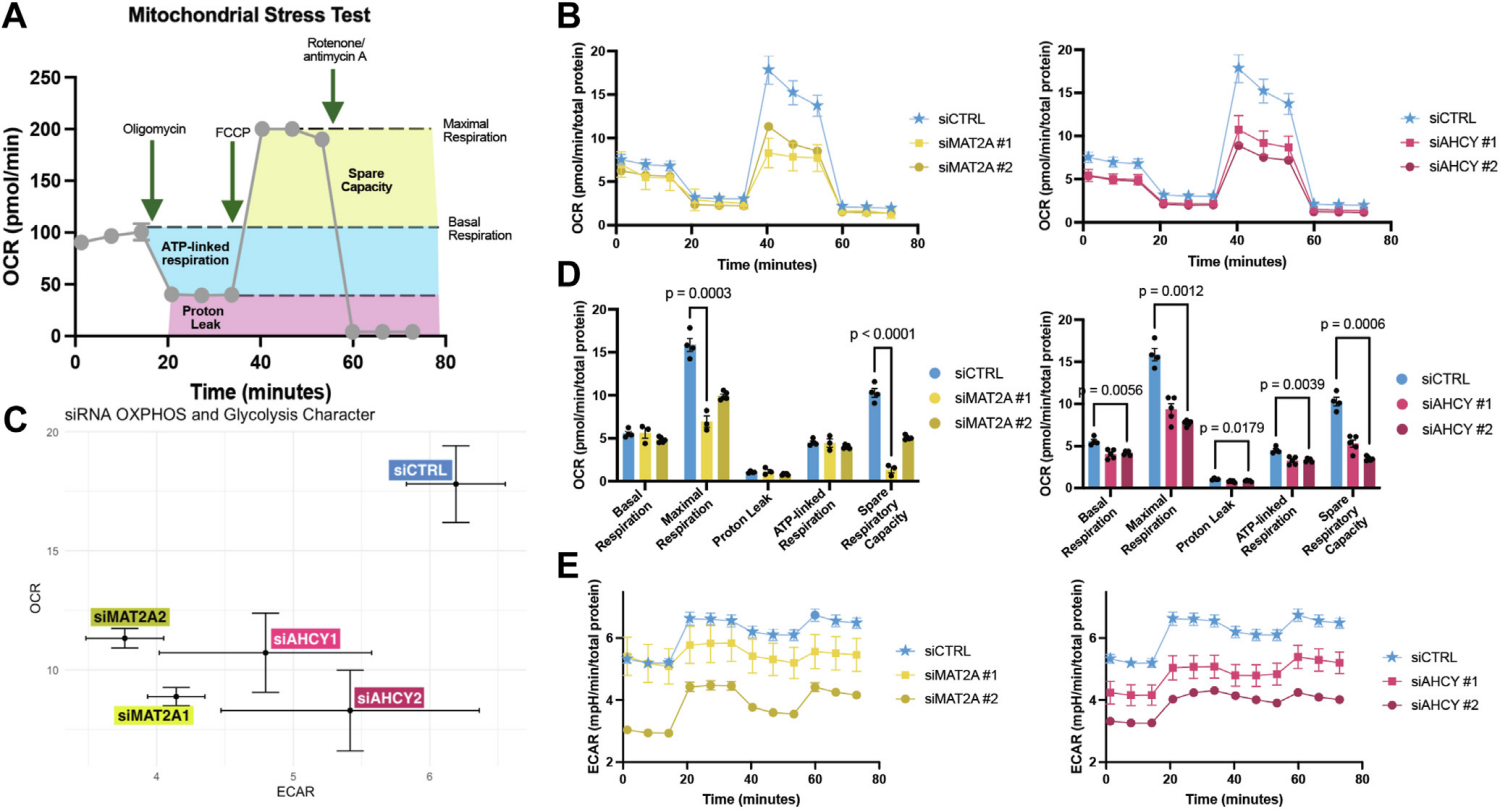
**Genetic knockdown of *MAT2A* and *AHCY* induces mitochondrial dysfunction in GBM.***A*, diagram of mitochondrial stress test indicating changes in oxygen consumption rate (OCR) following electron transport chain inhibitor compound injections. Highlighted regions correlate to respiratory parameters by calculated differences between OCR values. Individual values represent technical replicates. *B*, mitochondrial stress test OCR with LN229 cells with genetic knockdown of either *MAT2A* or *AHCY* 72 h post-transfection. *C*, energy map with mean maximal OCR and corresponding ECAR values of genetic knockdown LN229 cells, with *top right* indicating energetic, *bottom right* indicating glycolytic, *top left* indicating aerobic, and *bottom left* indicating quiescent phenotype. *D*, quantification of respiratory parameters for *MAT2A* and *AHCY* knockdown cells compared to control. Unpaired *t* test with Holm-Šídák correction was performed. *E*, mitochondrial stress test ECAR with LN229 cells corresponding to OCR measurements, with *top right* corner indicating a more energetically active state and *bottom left* corner indicating a more energetically inactive state.

We conducted the mitochondrial stress test (Fig. 3*A*) using the Seahorse bioanalyzer, which can measure oxidative metabolism. Cellular respiratory parameters can be calculated based on changes in dissolved oxygen and pH in live cell media. The readouts are translated to represent oxygen consumption rate (OCR) and extracellular acidification rate (ECAR), respectively. Following the addition of electron transport chain complex inhibitors (complex V inhibitor oligomycin (40) or complex I/III inhibitors rotenone/antimycin A^35^) or a proton uncoupler (trifluoromethoxy carbonyl cyanide phenylhydrazone, FCCP (41)) over a set time course during the experiment, we can uncover potentially impaired components of OXPHOS in knockdown cells. Given that *MAT2A* and *AHCY* are implicated in antioxidant production (16), we anticipated that silencing these genes would result in disrupted oxidative metabolism such that the deleterious effects of ROS accumulation would lead to reduced GBM survival.

Following FCCP injection, we found a diminished maximal OCR for both *MAT2A* and *AHCY* knockdown LN229 cells (Figs. 3*B*; S5*A*). This reflects a significant impairment of mitochondrial function and consequently reduced capacity to respond to energetic demands. In assessing maximal respiration rates and complementary ECAR, *MAT2A* knockdown showed significantly reduced rates (mean OCR 8.881 ± 0.383) compared to control (p < 1 × 10^−4^), as did *AHCY* knockdown (mean 10.721 ± 1.659, p = 4 × 10^−4^). Evaluating the maximal OCR and the coinciding ECAR together reveals a shift from a highly energetic state to an energetically inactive state (Figs. 3*C*; S5*D*). Compared to the control condition, both si*MAT2A*-LN229 and si*AHCY*-LN229 cells exhibited significantly reduced maximal respiration (p = 3 × 10^−4^, 1.2 × 10^−3^ respectively), spare respiratory capacity (p < 1 × 10^−4^, 6 × 10^−4^ respectively), and overall reduced glycolytic activity (Figs. 3, *D* and *E*; S5, *B* and *C*). This suggests that both *MAT2A* and *AHCY* are integral for preventing mitochondrial ROS overload to facilitate increased energy production and proliferation in GBM.

### MAT2a and AHCY inhibition impedes cell growth in GBM primary cells

To evaluate the effect of inhibiting the enzymatic activity of MAT2a and AHCY, we identified two commercially available selective inhibitors, AG-270 and aristeromycin, to use in further experiments. AG-270 is the first-in-class oral selective MAT2a allosteric inhibitor and is currently being tested in clinical trials for lymphoma and solid tumors (42). Aristeromycin is a naturally occurring compound first isolated from *Streptomyces citricolor* in 1968 (43). It demonstrates potent AHCY inhibition and antiviral activity (44), and although it has not advanced into clinical development, it has been investigated preclinically in prostate cancer with considerable efficacy (45).

**Figure 4:**
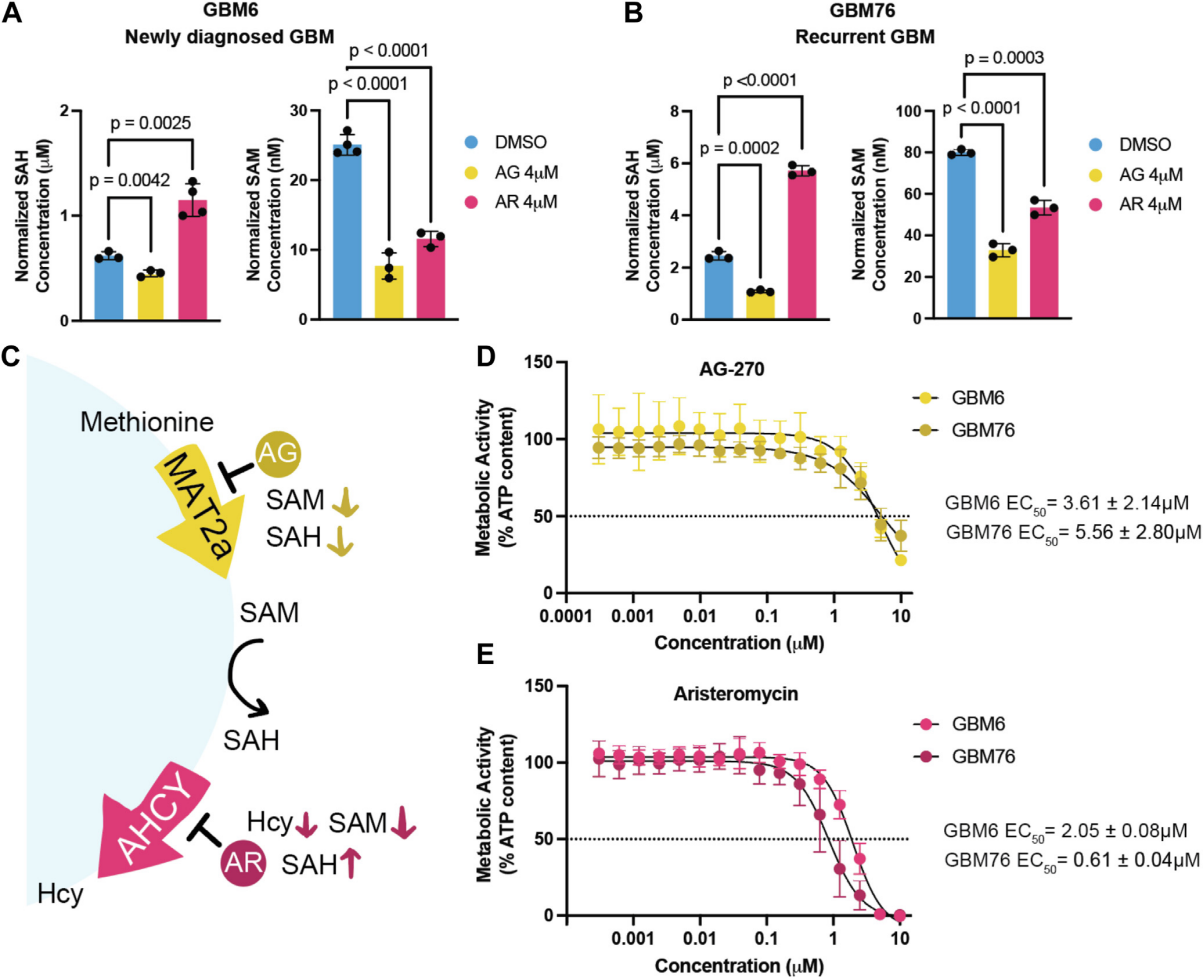
**Selective MAT2aand AHCY inhibitors reduce cell viability in GBM.***A* and *B*, SAH and SAM levels in patient-derived primary GBM cells 24 h after 4 μM of aristeromycin (AR), 4 μM of AG-270 (AG) treatment, or vehicle treatment. Individual values represent biological replicates. Shapiro–Wilk test and variance was compared using F test, followed by an unpaired two-tailed *t* test to assess differences between treatment groups. *C*, diagram of enzyme inhibition by each respective inhibitor compound and resulting changes in metabolite levels. *D* and *E*, dose-response curves for AG-270 (*top*) and aristeromycin (*bottom*) 72 h post-treatment with corresponding EC_50_ values in both GBM6 and GBM76 cells. ATP content was measured using CellTiterGlo to indicate cell viability.

GBM tumors are categorized into three molecular subtypes based on their transcriptional profile: classical, mesenchymal, or proneural (46). We treated multiple patient-derived xenograft (PDX) GBM cells designated as having a classical molecular subtype and one proneural cell type (47) (Table S1) using these two compounds individually (Fig. S6*A*). Two cell types, GBM6 and GBM76, were characterized as having a classical molecular subtype and were taken at initial diagnosis or upon tumor recurrence, respectively (47) (Table S1; Fig. S6, *B*–*E*). We were interested in analyzing classical PDX cells as they demonstrated the highest *MAT2A* and *AHCY* expression across subtypes based on RNASeq, Agilent-42502A, and HG-U133A datasets from The Cancer Genome Atlas (http://gliovis.bioinfo.cnio.es/) (48). Following 24-h treatment with AG-270, there was a significant reduction in SAM (SAM, p < 1 × 10^−4^ for both) as well as SAH (SAH, *p* = 4.2 × 10^−3^, 2 × 10^−4^ respectively) in both GBM6 and GBM76 compound-treated cells compared to vehicle control (Figs. 4, *A* and *B*; S6, *F* and *G*). Further, there was a significant decrease in SAM (p < 1 × 10^−4^, p = 3 × 10^−4^ respectively) and a significant increase in SAH (*p* = 2.5 × 10^−3^, p < 1 × 10^−4^ respectively) following aristeromycin treatment in either cell type (Figs. 4, *A* and *B*; S6, *F* and *G*). These changes in metabolite levels were expected considering these inhibitors are selectively targeting the enzymes responsible for producing or consuming these specific metabolites (Fig. 4*C*).

To study the effect of these compounds on cell viability, cells were treated with increasing concentrations of AG-270 or aristeromycin and evaluated for ATP levels after 72 h. Both treatments led to a dose-dependent reduction in ATP content with an EC_50_ value below 6 μM for AG-270 (3.61 ± 2.14 μM in GBM6; 5.56 ± 2.80 μM in GBM76) and 3 μM for aristeromycin (2.05 ± 0.08 μM in GBM6; 0.61 ± 0.04 μM in GBM76) in both cell types (Fig. 4, *D* and *E*). Only metabolically active cells are considered viable, so reduced ATP content is indicative of reduced cell viability. These results indicate that both AG-270 and aristeromycin effectively engage with their target enzyme and potently inhibit its function at low micromolar concentrations, leading to reduced GBM cell viability.

### MAT2a and AHCY inhibition compromises mitochondrial function in GBM primary cells

**Figure 5:**
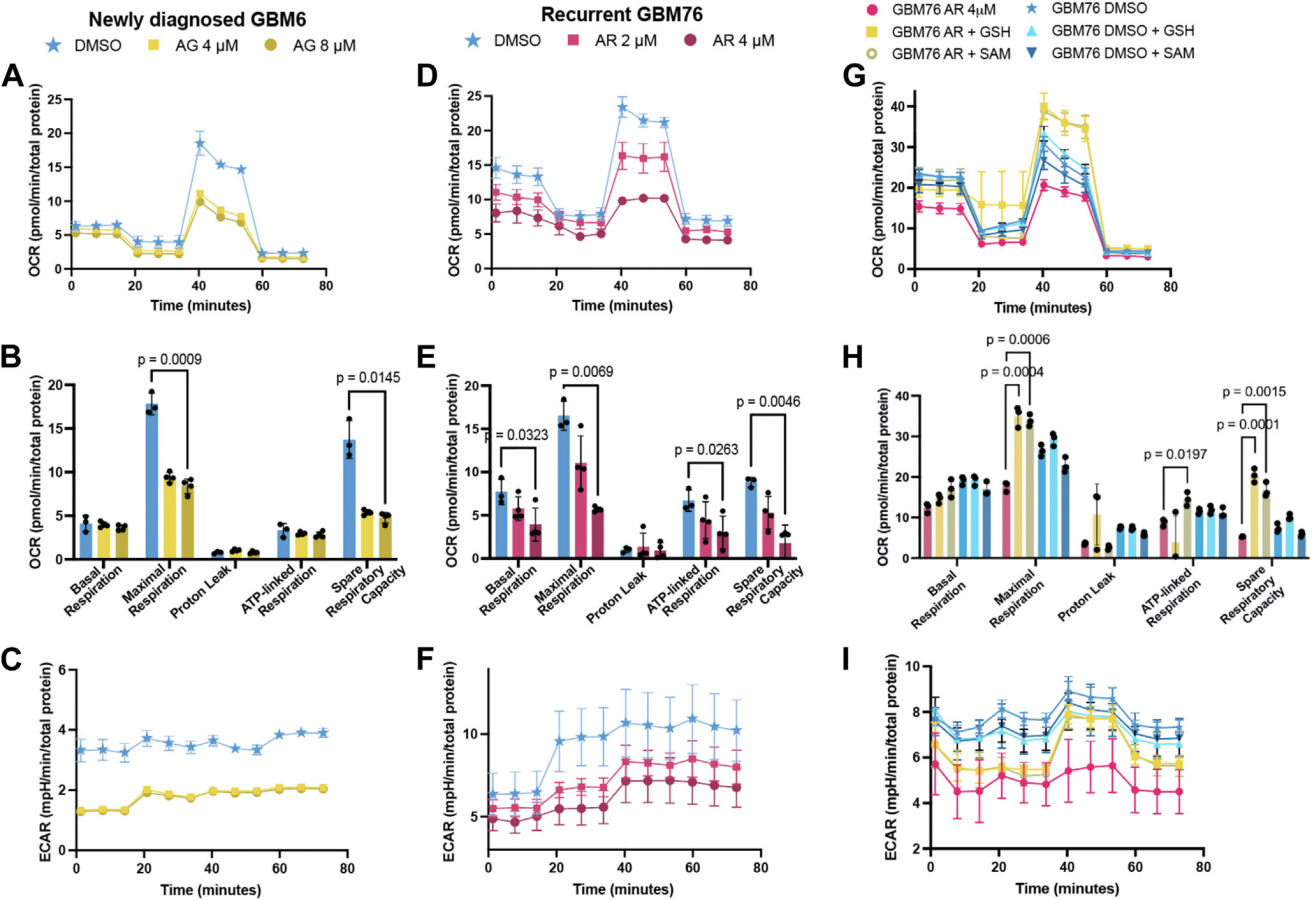
**MAT2aand AHCY enzyme inhibition disrupts mitochondrial function in GBM.***A* and *B*, mitochondrial stress test OCR with newly diagnosed GBM6 cells treated with 4 μM or 8 μM AG-270 or DMSO 24 h prior with corresponding quantification of respiratory parameters. Unpaired *t* test with Holm-Šídák correction was performed. *D* and *E*, OCR for recurrent GBM76 cells treated with 2 μM or 4 μM aristeromycin or DMSO 24 h prior with corresponding quantification of respiratory parameters. *C* and *F*, mitochondrial stress test ECAR corresponding to OCR measurements. *G* and *H*, mitochondrial stress test experiment with or without aristeromycin supplemented with or without glutathione (GSH) or S-adenosylmethionine (SAM) treated 24 h prior with corresponding quantification of respiratory parameters. Unpaired *t* test with Holm-Šídák correction was performed. *I*, mitochondrial stress test ECAR corresponding to OCR rescue experiment.

We sought to discover the perturbations in oxidative metabolism associated with MAT2a or AHCY inhibition. We again conducted the mitochondrial stress test to evaluate mitochondrial respiration following short-term (24-h) inhibitor treatment using the Seahorse bioanalyzer. AG-270 significantly reduced cellular respiration in GBM6 cells, with lower maximal respiration (p = 9 × 10^−4^) and spare respiratory capacity (*p* = 0.0145) (Figs. 5, *A* and *B*; S8, *A* and *B*). Aristeromycin significantly reduced multiple parameters of cellular respiration in GBM76 cells, including basal respiration (*p* = 0.0323), maximal respiration (*p* = 6.9 × 10^−3^), ATP-linked respiration (*p* = 0.0263), and spare respiratory capacity (*p* = 4.6 × 10^−3^) (Figs. 5, *D* and *E*; S8, *D* and *E*). In both cases, the inhibitors also reduced glycolytic activity according to the extracellular acidification rate relative to vehicle control (Figs. 5, *C* and *F*; S8, *C* and *F*). These results indicate that MAT2a or AHCY inhibition significantly reduces cellular respiration and mitochondrial function in GBM.

To further characterize the MAT2a and AHCY pathway in GBM, we performed a rescue experiment by supplementing inhibitor treatment with glutathione and SAM. We found that glutathione or SAM both rescued GBM76 cellular respiration from AHCY inhibitor treatment with increased maximal respiration (p = 4 × 10^−4^; p = 6 × 10^−4^, respectively) and spare capacity (p = 1 × 10^−4^; *p* = 1.5 × 10^−3^, respectively; Figs. 5, *G*–*I*; S9, *A*–*E*). These results suggest that redox metabolism is especially sensitive to AHCY inhibition in GBM76 cells and is critical for GBM cell growth and survival.

### MAT2a and AHCY inhibition reduces antioxidant production and induces oxidative stress in GBM

**Figure 6:**
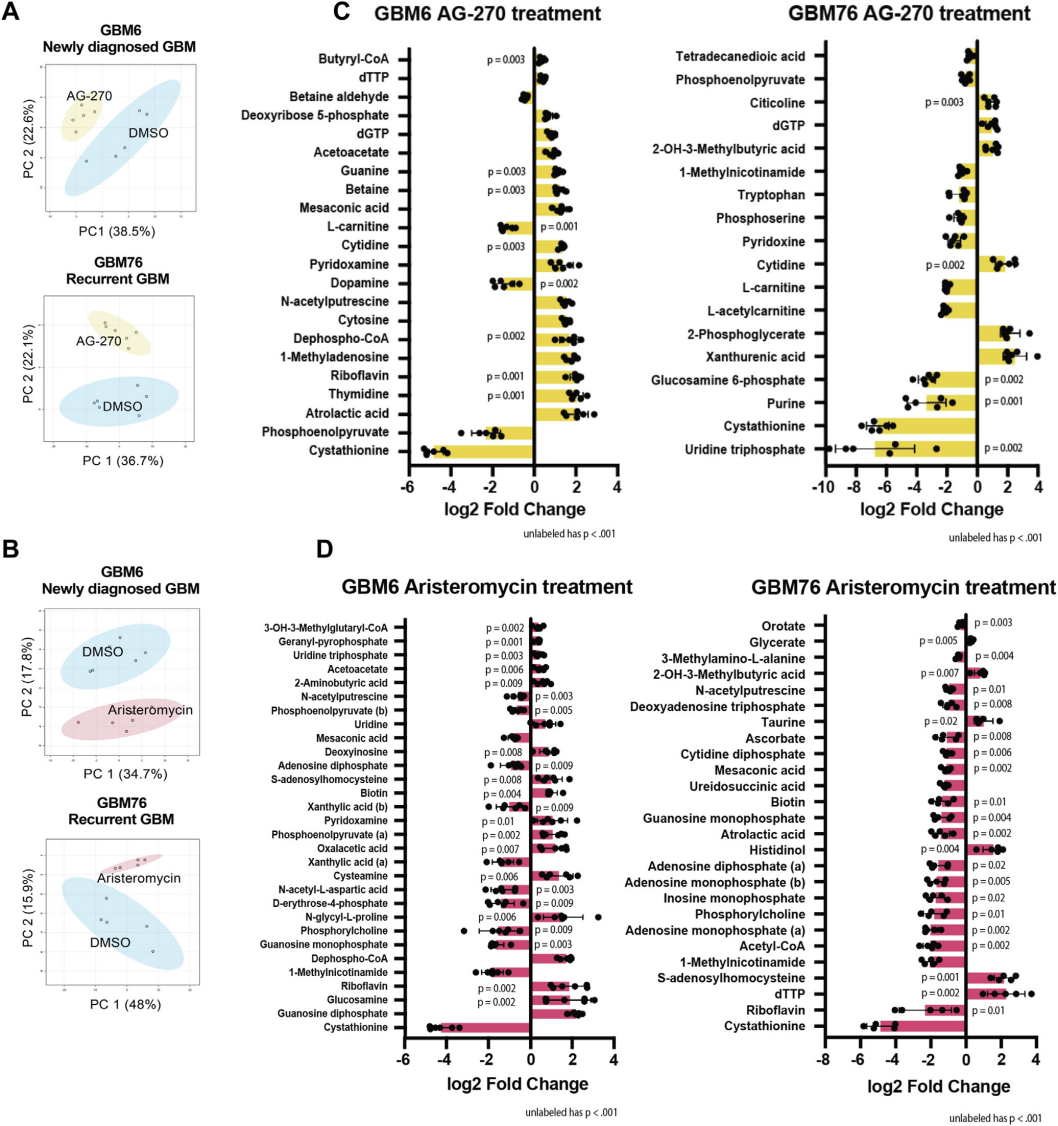
**GBM exhibits oxidative stress and compromised lipid metabolism upon MAT2aand AHCY inhibition.***A* and *B*, principal component analysis of GBM6 and GBM76 cells grown in serum-free media comparing DMSO to 4 μM AG-270 (*A*) or 4 μM aristeromycin (*B*) treatment. Cells were analyzed 24 h post-treatment by LC-MS. Sample peak intensities were normalized using log transformation and Pareto scaling. *C* and *D*, significantly reduced and increased metabolites in GBM6 and GBM76 cells treated with AG-270 (*C*) and aristeromycin (*D*) compared to control using unpaired *t* test with a *p*-value of less than 0.05. (*B*) indicates the alkaline metabolite while (*A*) indicates the acidic metabolite upon ionization. Individual values represent biological replicates.

To characterize the metabolic profile of primary GBM cells following inhibitor treatment, we conducted targeted metabolomic analysis to quantitatively measure specific groups of metabolites in these samples and potentially uncover novel associations between metabolite levels and the respective treatment conditions for GBM6 and GBM76 cells (49). We wanted to assess how inhibition of either MAT2a or AHCY impairs oxidative metabolism. We used a panel of 366 biochemically annotated one-carbon metabolites and TCA cycle metabolites as our internal standard to identify the metabolites within our samples. This panel allowed us to measure not only metabolites that are directly involved in the methionine cycle but also those that are broadly involved in one-carbon metabolism-nucleotide biosynthesis, lipid metabolism and transport, polyamine synthesis, B vitamin metabolism, neuroprotective programs (50), and the TCA cycle, highlighting key factors for mitochondrial function (51). To ensure that serum supplementation would not artificially influence cellular metabolism and thus skew intracellular metabolite levels, we cultured GBM6 and GBM76 cells using serum-free media for our metabolomics analysis. Groups were evaluated using principal component analysis and were found to cluster by treatment condition (Figs. 6, *A* and *B*; S11*A*). Both GBM6 and GBM76 cells treated with aristeromycin demonstrated a significant increase in SAH (log2FC 0.98, *p* = 7.5 × 10^−3^ for GBM6; log2FC 2.17, *p* = 1.4 × 10^−3^ for GBM76, Figs. 6*D*; S11*B*), as expected from previous analysis (Figs. 4, *A* and *B*; S6, *F* and *G*). Several other metabolites were significantly reduced or increased commonly across different treatment groups and cell types (Fig. S12, *A*–*C*; Table S2).

One metabolite in particular that is downstream of methionine metabolism is cystathionine, a sulfur-containing molecule, which is implicated in redox homeostasis as a substrate for glutathione synthesis (52). Cystathionine was markedly depleted in all treatment groups (log2FC −4.82, *p* = 2.02 × 10^−6^ for GBM6 AG-270; log2FC −4.28, *p* = 3.01 × 10^−7^ for GBM6 aristeromycin; log2FC −6.56, *p* = 9.13 × 10^−6^ for GBM76 AG-270; log2FC −4.85, p = 6 × 10^−4^, Figs. 6, *C* and *D*; S12, *A* and *B*). The remaining top significant metabolites that were reduced following AG-270 treatment in both cell types were L-carnitine (log2FC −1.33, *p* = 1.2 × 10^−3^ for GBM6; log2FC −2.02, *p* = 6.27 × 10^−5^ for GBM76), which transports long chain fatty acids into the mitochondria to be oxidized for energy (53) and is associated with ROS scavenging and enhancing antioxidant capacity (54) and phosphoenolpyruvate (log2FC −2.31, p = 4 × 10^−4^ for GBM6; log2FC −0.76, p = 6 × 10^−4^ for GBM76), a high-energy metabolic intermediate that supports cancer proliferation and balances ROS levels (55, 56) (Figs. 6*C*, S12*A*). Among the metabolites that were increased in both cell types following AG-270 treatment were cytidine (log2FC 1.33, p = 3 × 10^−3^ for GBM6; log2FC 1.84, *p* = 1.6 × 10^−3^ for GBM76), a nucleoside and an important substrate for mitochondrial biogenesis (57), and cholesteryl sulfate (log2FC 1.96, *p* = 0.0158 for GBM6; log2FC 1.12, *p* = 0.020 for GBM76), which inhibits cholesterol synthesis (58) and upregulates antioxidant capacity in normal astrocytes (59), and 1-methyladenosine (log2FC 1.79, *p* = 6.01 × 10^−5^ for GBM6; log2FC 1.12, *p* = 3.6 × 10^−3^ in GBM76), which behaves as an mRNA modification that is upregulated during neuronal oxidative stress damage (60) (Figs. 6*C*, S12*A*).

The remaining metabolites that were significantly reduced upon aristeromycin treatment in both cell types included phosphorylcholine (log2FC −1.55, *p* = 8.9 × 10^−3^ for GBM6; log2FC −1.80, *p* = 0.0144 for GBM76), which is involved in lipid homeostasis and associated with GBM proliferation (61), 1-methylnicotinamide (log2FC −1.83, *p* = 7.75 × 10^−6^ for GBM6; log2FC −2.04, *p* = 4.17 × 10^−6^ for GMB76), a downstream product of methionine metabolism that coordinates energy metabolism (62), guanosine monophosphate (log2FC −1.53, *p* = 0.0225 for GBM6; log2FC −1.39, *p* = 4.1 × 10^−3^ for GMB76), a precursor for vasodilation and neurotransmission signaling and is associated with recurrence in GBM (63), mesaconic acid (log2FC −0.84, *p* = 9.54 × 10^−5^ for GBM6; log2FC −1.12, *p* = 2.1 × 10^−3^ for GMB76), a fatty acid associated with reduced inflammatory response in the brain (64), and N-acetylputrescine (log2FC −0.62, *p* = 2.9 × 10^−3^ for GBM6; log2FC −0.95, *p* = 0.0138 for GMB76), which is involved in fatty acid oxidation and is higher in GBM patients than controls (65) (Figs. 6*D*, S12*B*). Other than SAH, oxaloacetic acid (log2FC 1.17, *p* = 6.7 × 10^−3^ for GBM6; log2FC 0.45, *p* = 0.0320 for GBM76), shown to promote brain mitochondrial biogenesis and reduce neuroinflammation (66), and glycerate (log2FC 0.23, *p* = 0.0305 for GBM6; log2FC 0.32, *p* = 4.5 × 10^−3^ for GBM76), a product of glycerol oxidation that acts as a metabotoxin at sufficiently high levels (67), were the two significantly upregulated metabolites for both cell types treated with aristeromycin (Fig. S12*A*). 3-Hydroxy-3-methylglutaryl-CoA, the metabolite whose conversion initiates cholesterol synthesis (68), was selectively increased in GBM6 cells treated with Aristeromycin (log2FC 0.36, *p* = 0.0016, Fig. 6*D*). GBM76 cells treated with Aristeromycin had significantly reduced levels of alkaline biotin and riboflavin (Fig. S12*B*). Biotin and riboflavin are important B vitamins that facilitate carboxylation and redox reactions in the brain, respectively (69). Riboflavin is critical for mitochondrial aerobic respiration while biotin is necessary for mitochondrial fatty acid oxidation and gluconeogenesis (70). A previous study highlighted that biotinylation is an important modification in glioma stem cells and disrupted biotin distribution leads to cholesterol depletion, impaired OXPHOS, disrupted GBM proliferation, and reduced invasiveness (71). This indicates that this post-transcriptional modification may be diminished in GMB76 cells and upregulated in response to GBM6 methionine pathway inhibition. L-carnitine was more abundant in GMB6 cells at baseline (Fig. S10*C*) and was significantly depleted in both cell types treated with AG-270 (Fig. S12*B*). GBM relies on L-carnitine for antioxidant enzyme activity (72); it is more highly abundant in both newly diagnosed and recurrent GBM compared to normal brain tissue (73); and supplementation mitigates cell death from TMZ or hydrogen peroxide (73). Collectively, these results suggest that MAT2a or AHCY inhibition compromises GBM cellular metabolism by disrupting antioxidant production, fatty acid transport, cell membrane integrity, nucleotide synthesis, and other related protumorigenic programs.

## Discussion

Glioblastoma remains an incurable disease with limited treatment options. Our study broadly investigated the selective inhibition of methionine metabolism to target GBM progression. Methionine is converted into universal methyl donor SAM by MAT2a. Upon donating its methyl group, SAM is converted to SAH and is hydrolyzed by AHCY to generate homocysteine to be recycled or to synthesize glutathione. We anticipated inhibiting one of these steps would compromise GBM cell survival by hindering glutathione production. We found that siRNA-mediated depletion and pharmacologic inhibition of MAT2a or AHCY hindered GBM cell survival. Inhibitor-treated primary GBM cells exhibited increased pro-oxidative metabolites, reduced antioxidative metabolites, and compromised energy production. These results suggest that restricting methionine metabolism *via* blocking either enzyme reduces GBM cell growth and is implicated in regulating antioxidant capacity in GBM.

The brain is responsible for 20% of total oxygen consumption, which is likely why brain tissue is particularly sensitive to oxidative stress and antioxidant capacity is critical for preventing oxidative damage (74). ROS production begins with molecular oxygen during incomplete reduction in the mitochondrial respiratory chain. These ROS participate in redox signaling and can also facilitate tumorigenic programs (75), but excessive ROS accumulation due to deficient antioxidant neutralization results in oxidative damage (76). GBM tumors are prone to increased ROS generation as they are more metabolically active than healthy brain tissue and face comparably limited vascularity (77). The accumulation of ROS and oxidative stress directly impacts mitochondrial function and cellular bioenergetics. Therefore, we evaluated both oxidative stress and cellular respiration in the context of genetic knockdown. We see a baseline level of lipid peroxidation in control LN229 cells, but this is greatly increased upon either *MAT2A* or *AHCY* knockdown, indicating a direct relationship between methionine metabolism and GBM ROS:antioxidant homeostasis. We then followed a protocol for the mitochondrial stress test that assesses mitochondrial function to determine the persistent effects of oxidative stress on the bioenergetic profile (78). We observed reduced rates of oxygen consumption, which is reflective of compromised OXPHOS and mitochondrial function. Collectively, these results demonstrate the importance of methionine metabolism in preventing GBM oxidative damage.

Previous studies have demonstrated how GBM is highly sensitive to redox state disruption by other small molecules. TMZ induces oxidative stress by oxidative removal of methyl groups from DNA and inducing DNA damage (79); chloroquine increases ROS production by interfering with lysosomal function and cellular debris clearance (80); curcumin can behave as a pro-oxidant in GBM and mitigate cell invasion (81, 82); menadione/ascorbate combination treatment directly interferes with mitochondrial electron transport and leads to ROS accumulation (83). However, these compounds either demonstrate off-target effects leading to adverse events that harm normal brain tissue or are susceptible to metabolic neutralization, resulting in poor stability (83, 84, 85). The benefit of selectively targeting a metabolic enzyme that is directly involved in antioxidant production limits the risk of nonspecific action. AG-270 demonstrated superior *in vivo* metabolic stability across species (36) while aristeromycin and other adenosine analogs appear to have a longer half-life than endogenous adenosine (86). It is unclear whether AG-270 modifies SAM-dependent enzyme methylation capability. Aristeromycin was not brought into clinical studies due to possible *in vivo* toxicity, but we used it as a tool compound with demonstrated potent AHCY inhibition as there are other derivatives that have been developed since its discovery with reduced toxicity. Further research is still needed to determine the brain penetrance of each of these compounds and their therapeutic potential in combination with standard-of-care therapy in GBM.

A recent study identified the downstream methionine substrate cystathionine as the most highly abundant metabolite in the invasive tumor edge fraction compared to the tumor core fraction, implicating antioxidant metabolism in GBM invasion (32). In our study, cystathionine was most depleted consistently across all treatment groups. This suggests that reduced GBM cell viability and perhaps reduced invasive capacity are associated with MAT2a or AHCY inhibition. We did not investigate the invasiveness of GBM cells following genetic knockdown or enzyme inhibition, so further studies are needed to confirm how MAT2a or AHCY inhibition impacts invasion in GBM.

SAM has been shown to behave as an antioxidant by inhibiting lipid peroxidation in ischemic brain tissue *in vitro* and was correlated with higher levels of glutathione in all brain regions measured (87). This study also indicated that SAM reveals a stronger antioxidant effect in the brain during oxidative stress that is less extensive under normal conditions, and SAM led to improved mitochondrial function (87). In our study, supplementation with either glutathione or SAM in combination with drug treatment both rescued mitochondrial function, evidenced by restored cellular respiration. Thus, when MAT2a or AHCY inhibition results in significantly reduced SAM levels, it is possible that this is directly associated with diminished mitochondrial function. Unraveling SAM utilization in GBM is clinically relevant for improving treatment strategies for GBM. The methionine pathway appears to be a significant vulnerability for other reasons beyond redox capacity. Methylthioadenosine phosphorylase is responsible for the methionine cycle salvage pathway, and cells display increased sensitivity to methionine depletion without it (88). While methylthioadenosine phosphorylase deficiency has been identified across cancers, homozygous deletion occurs in approximately half of all GBM tumors (89) and is not associated with elevated methylthioadenosine (90). Interestingly, researchers have identified a negative correlation between DNA methylation and brain tumor grade, where DNA hypomethylation is more common in GBM patients having reduced 5-methylcytosine content (91). Understanding the widespread implications of targeting MAT2a and AHCY in combination with standard-of-care treatment beyond antioxidant capacity and metabolic function is necessary for refining GBM therapeutic strategies.

While we highlight the importance of our work in GBM, we also acknowledge the limitations of this study. We analyzed six samples per treatment group for our metabolomics analysis, which made identifying the significance of differentially abundant metabolites more challenging. As such, we did not see a significant difference in reduced or oxidized glutathione between treatment groups. Despite this, our analysis still revealed significantly diminished cystathionine levels following MAT2a and AHCY inhibition. We used two PDX primary cells as opposed to established GBM cell lines. GBM76 cells were taken from a patient who was diagnosed at age 37 and underwent treatment with radiotherapy, TMZ, and EGFR inhibitor erlotinib prior to biopsy collection (47). GBM6 cells were taken from a patient upon initial diagnosis at age 65 (47). While these two PDX models are not generalizable to all tumors, they are a superior model than established cell lines typically used in previous studies as they more closely resemble the original tumor at both phenotypic and molecular levels. Another limitation is that our experiments were in *vitro* and thus did not consider the implications of treatment in the context of multiple different cell types and complex interactions within the native tumor microenvironment. Accurately modeling the GBM tumor microenvironment is challenging and is an evolving area of research in the field. However, despite the limitations, our findings help elucidate the mechanistic basis of methionine restriction in GBM treatment.

In conclusion, targeting methionine metabolism *via* MAT2a or AHCY inhibition is a possible avenue to arrest cancer progression and improve outcomes for GBM patients. We found that targeting these enzymes leads to compromised antioxidant capacity, reduced mitochondrial function, and cell death, as evidenced by reduced cellular respiration and reduced levels of antioxidant markers. Future studies are needed to better elucidate this mechanism and to develop clinical candidates for these proteins with appropriate efficacy and safety profiles. This study highlights how targeting methionine metabolism impedes GBM redox capacity and informs clinical studies for improved therapeutic strategies.

## Experimental procedures

### Cell lines

The human glioblastoma cell line LN229 was purchased from ATCC. Patient-derived xenograft cells were acquired from Dr Jann Sarkaria’s laboratory (Mayo Clinic). Briefly, cells were cultured in cell culture–treated flasks (CellTREAT) in Dulbecco’s modified Eagle’s medium and Ham’s F-12 nutrient mixture (F12) (Gibco; Thermo Fisher Scientific, Inc., cat. no. 11320033) supplemented with 10% fetal bovine serum (Gibco, Thermo Fisher Scientific, Inc., cat. no. A5256801) and 100U penicillin and 100 mg/ml streptomycin (Gibco, Thermo Fisher Scientific, Inc., cat. no. 10099141). Serum-free stem cells were maintained in DMEM:F12 supplemented with neuronal cell supplement (StemCell Technologies, cat. no. 05711), 200 mM L-glutamine (Corning, cat. no. 25005CI), human FGF and human EGF supplement (Thermo Fisher Scientific, Inc., cat. nos. PHG0261 & PHG0311), as well as penicillin/streptomycin (Thermo Fisher Scientific, Inc.). All cultures were maintained in a humidified incubator at 37 °C with 5% CO_2_.

### Antibodies and siRNA constructs

MAT2a antibody (cat. no. PA5-115550) and AHCY antibody (cat. no. MA5-42797) were purchased from Thermo Fisher Scientific. An ON-TARGET Plus Human siRNA smartpool (Horizon Discovery) along with two Silencer Validated siRNAs (Invitrogen, Thermo Fisher Scientific) for each gene of interest were purchased. For siRNA #1 of both genes, ON-TARGET Plus Human siRNA, 5 nmol containing three pre-selected siRNA constructs with guaranteed silencing at the mRNA level 48 h after transfection using 100 nM siRNA was used. The single target accession of *MAT2A* smartpool siRNA is NM_005911.6, while the smartpool siRNA for *AHCY* targets 13 accessions. For siRNA #2 of both genes, Silencer Pre-Designed siRNA for *MAT2A* (ID# 111626) with NM_005911.5 for the interrogated sequence was used, while the siRNA for *AHCY* (ID# s1195) with five interrogated sequences was used. These targeted sequences can be accessed on the respective websites for these constructs.

### DepMap analysis

Methionine pathway genes perturbation effects were captured by generated CRISPR Chronos gene dependency scores associated with a significant *p*-value for glioblastoma, diffuse glioma, and CNS tumor lineages. These scores and *p*-values were collected and sorted for each cell lineage, and effect scores were averaged for each gene target. Statistical significance of the gene effect was defined as p = value < 0.0005, as set by DepMap (17).

### siRNA transfection

LN229 cells were seeded in 6-well plates at a density of 2.4 × 10^5^ cells per well (or half for a 96-h post-transfection incubation experiment). After 24 h, once they had reached 70% confluency, cells were transfected with 100 nM of each siRNA using Lipofectamine RNAiMax reagent (Invitrogen; Thermo Fisher Scientific, Inc., cat. no. 13778150). After 48 h, cells were collected for RNA extraction, protein extraction, or flow analysis.

### Western blot

Cell lysates were generated by mechanical disruption using lysis buffer (Cell Signaling Technology, cat. no. 9803) with a protease inhibitor cocktail (Roche, Millipore Sigma, cat. no. 4693124001) and phosphatase inhibitor cocktail (Sigma Aldrich, cat. no. P5726) in deionized water. Electrophoresis was performed using a precast SDS-PAGE gel, electrophoresis chamber and power supply for 120 min, and subsequent semidry transfer with the Trans-Blot Turbo Transfer System and reagents (BioRad Laboratories, Inc.). Membrane was stained with Ponceau Stain, washed with TBST, then blocked for 5 minutes with EveryBlot blocking buffer (BioRad, cat. no. 12010020). The blot was then incubated in primary antibody solution for 1.5 h, washed three times with TBST, incubated in secondary antibody solution for 1.5 h, then washed another three times and imaged using the ChemiDoc MP Imaging System (BioRad Laboratories, Inc.). Blots were quantified using ImageJ and normalized to siCTRL band intensity. Statistical significance was assessed using unpaired parametric t-tests with Holm-Šídák multiple comparisons test.

### Flow cytometry

LN229 cells were transfected at 0 h and the media was replaced after 72 h. Ninety six hours post-transfection, media was added back to the respective wells and BODIPY (Thermo Fisher Scientific, Inc., cat. no. D3922) was added directly to the well at a concentration of 2 μM. Cells were incubated for 15 min at 37 °C and protected from light. Cells were collected and centrifuged along with floating cells in the media and were washed with PBS. Cells were stained with SYTOX Blue and prepared for flow analysis by the Georgetown University Core Facility. Intact cells were gated for final analysis and normalized to peak intensity and number of events per sample.

### Mitochondrial stress test

Cells were plated in a Seahorse microplate at a density of 1.8 × 10^4^ cells per well for 4 to 8 h. Cells were subsequently treated with different concentrations of AG-270 and aristeromycin or DMSO vehicle treatment. After 24 h, the cell media was exchanged with Seahorse assay media, and compound was added to the ports: 1.5 μM oligomycin in port A, 1 μM FCCP in port B, and 0.5 μM rotenone and antimycin A in port C, with port D empty. Cells were kept in a non-CO2 incubator at 37 °C for an hour before beginning the assay. Using the Seahorse XFe96 bioanalyzer (Agilent Technologies), OCR and ECAR were measured on an interval after each injection to obtain 12 readings for each well over the course of the experiment. The injection compounds were used from the mitochondrial stress test kit (Agilent) along with the corresponding protocol. In brief, cells were measured at baseline, then 1 μM oligomycin was injected into each well to inhibit ATP synthase, so the difference from baseline reflects oxygen consumption due to ATP respiration. Then 1.5 μM FCCP was injected into each well, which is a proton uncoupler to allow maximal respiration, and therefore the difference from baseline reflects the spare respiratory capacity of the cells. Finally, 0.5 μM rotenone and antimycin A were added to inhibit complex I and III, which extinguishes cellular respiration completely to reflect proton leakage in the electron transport chain. Each experiment was normalized to total protein in each well using the Pierce BCA protein assay kit (Thermo Fisher Scientific, Inc.). The statistical significance of differences between groups was evaluated using an unpaired parametric *t* test with Holm-Šídák multiple comparisons test.

### Metabolic activity viability assay

CellTiter-Glo Luminescent Cell Viability Assay kit (Promega) was used to determine the number of viable cells following inhibitor compound treatment. Cells were plated in white bottom 96-well plates at a density of 3 × 10^4^ cells per well in 100 μl complete media. Each plate was treated with a serial dilution of 16 concentrations, along with a negative control (0.2% DMSO or less) and a positive control (Bortezomib). After 72 h, plates were removed from incubation and brought to ambient temperature for 30 min. Hundred microliters of reagent was then added to each well, the plate was shaken for 30 s to mix thoroughly, then incubated for 10 min before acquiring measurements using the CLARIOstar Plus Microplate Reader.

### Targeted metabolomics

All LC-MS grade solvents including acetonitrile and water were purchased from Fisher Optima grade, Fisher Scientific. High purity formic acid (99%) was purchased from Thermo-Scientific. Debrisoquine and 4-nitrobenzoic acid and 2-hydroxyglutarate (2-HG) were purchased from Sigma-Aldrich. Targeted analysis was performed by the Georgetown University Core Facility. Samples were prepared and run using the QTRAP 5500 LC-MS/MS System (Sciex) to quantitate 332 endogenous molecules. Results were normalized to internal standards and processed using MultiQuant 3.0.3 (Sciex).

### Metabolomics enrichment analysis

Pairwise comparisons between vehicle and small molecule treatment for both cell types was performed using Metaboanalyst software. Using single-factor statistical analysis, we normalized by logarithmic data transformation and Pareto scaling. All figures were subsequently generated including the principal component analysis plot, volcano plot, and heatmap. Statistical significance of differences between treatments was assessed using two-sample t-tests.

### Immunoassays for metabolite detection

The experiments were conducted in accordance with each respective manual provided using approximately 1 × 10^6^ cells per replicate. The SAH (cat. no. MET-5151) and SAM (cat. no. MET-5152) ELISA Kits were both purchased from Cell Biolabs, Inc. Each experiment was normalized to total protein in each sample using the Pierce BCA Protein Assay Kit (Thermo Fisher Scientific, Inc.). Normality in metabolite measurements was tested using the Shapiro–Wilk test and variance was compared using F test, followed by an unpaired two-tailed *t* test to assess differences between treatment groups.

### Cell metabolomics using QTRAP 5500

Targeted metabolomics method was used to quantitate >450 endogenous metabolites using QTRAP 7500 LC-MS/MS System (Sciex). For the purpose, 100 μl of extraction buffer (methanol/water 50/50) containing 200 ng/ml of debrisoquine as internal standard for positive mode and 200 ng/ml of 4-nitrobenzoic acid as internal standard for negative mode was added to the cell pellet and sample tube was plunged into dry ice for 30 s and 37 °C water bath for 90 s. This cycle was repeated for two more times and then samples were sonicated for 1 min. The samples were vortexed for 1 min and kept on ice for 20 min followed by the addition of 100 μl of acetonitrile. The samples were incubated at −20 °C for 20 min for protein precipitation. The samples were centrifuged at 13,000 rpm for 20 min at 4 °C. The supernatant was transferred to MS vial for LC-MS analysis. Twenty microliters of each prepared sample was mixed to generate the pooled QC sample.

### NIST plasma sample preparation

Twenty five microliters of NIST plasma sample was dissolved in 100 μl of extraction buffer (methanol/water 50/50) containing 200 ng/ml of debrisoquine as internal standard for positive mode and 200 ng/ml of 4-nitrobenzoic acid as internal standard for negative mode. The sample was vortexed for 30 s and incubated on ice for 20 min followed by addition of 100 μl of acetonitrile and incubation at −20 °C for 20 min. Samples were centrifuged at 13,000 rpm for 20 min at 4 °C. The supernatant was transferred to MS vial for LC-MS analysis.

One microliter of the prepared sample was injected onto a Kinetex F5, 2.6 μm 100 Å 150 × 2.1 mm (Phenomenex) using SIL-30 AC auto sampler (Shimazdu) connected with a high flow LC-30AD solvent delivery unit (Shimazdu) and CBM-20A communication bus module (Shimazdu) online with QTRAP 7500 (Sciex) operating in negative ion mode. A binary solvent comprising of water with 0.1% formic acid (solvent A) and acetonitrile with 0.1% formic acid (solvent B) was used. The extracted metabolite was resolved at 0.2 ml/min flow rate. The LC gradient conditions were as follows: initial – 100% A, 0% B for 2.1 min; 14 min – 5% A, 95% B till 15 min; 15.1 min – 100% A, 0% B till 20 min. The auto sampler and oven were kept at 15 °C and 30 °C, respectively. Source and gas setting for the method were as follows: curtain gas = 40, CAD gas = 9, ion spray voltage = 1700 V in positive mode and ion spray voltage = 1600 V in negative mode, temperature = 350 °C, ion source gas 1 = 30, and ion source gas 2 = 50.

### Data processing

The data were normalized to respective internal standard area and processed using MultiQuant 3.0.3 (Sciex). The quality and reproducibility of LC-MS data was ensured using a number of measures. The column was conditioned using the pooled QC samples initially and were also injected periodically to monitor shifts in signal intensities and retention time as measures of reproducibility and data quality of the LC-MS data. We also ran NIST plasma sample, periodically, prepared in the same manner to check the instrumental variance. We also have blank solvent runs between set of samples to minimize carry-over effects. The report of pooled QC and NIST plasma is provided in excel sheet attached.

### Statistical analysis

Dose-response curve data is represented in the form of mean ± SEM. All other data is represented in the form of mean ± SD. GraphPad Prism Software version 10.2.1 was utilized to perform all regression analysis, paired t-tests, and one-way ANOVA. All experiments were conducted three times using three or more technical replicates or in agreement with assay instructions for statistical power.

## Data availability

The data generated in this study are available upon reasonable request from the corresponding author.

## Supporting information

This article contains supporting information.

## Conflict of interest

The authors declare that they have no conflicts of interest with the contents of this article.

## Supporting information

All Seahorse Data

Comparisons between metabolite groups

Compound Data

Experimental Statistics

Fold change metabolite calculations

GBM6_analysis

GBM76_analysis

Metabolomics data

Supporting Information Figures Updated

Supporting Information Tables

Targeted metabolomics panel
